# Martyred Pyhsicians

**Published:** 1878-10-20

**Authors:** 


### MARTYRED PHYSICIANS.

Those physicians who have nobly remained at their post to battle with the yellow fever scourge, and those who have volunteered from a distance to visit the yellow fever districts, may well be styled martyrs to science and to humanity. We have gathered from our exchanges the following list of names who have fallen victims to the disease. Honor to their memory and peace to their souls! W.

Port Gibson; Dr W. D. Spratt.
Grand Junction; Dr. J. H. Prewitt.
Austin, Texas; Dr. Manning.

Memphis; Dr. F. Sarner, Dr. Hopson, Dr. K. L. Watson, Dr. L. R. Laske, Dr. W. R. Hodges, Dr. S. M. Dickinson, Dr. R. B. Williams, Dr. Mead, volunteer from Ky., Dr. J. B. Woodward, Dr. McGregor, Dr. T. L. Bond, vol., Dr. Menees, vol., Dr. J. Runner, of Ind., Dr. John Erskine, Dr. J. E. Penn, Dr. J. B. Hicks, Dr. J. S. Bankson, Dr. E. A. Cheeves, Dr. Paul Otey, Dr. E. T. Easley, vol., Dr. J. M. S. White, vol., Dr. W. K. Lowry, Dr. E. P. White, Dr. J. F. Sample, Dr. Force, volunteer, Dr. B. W. Avant.

Vicksburg.—Dr. Glass, Dr. Booth, Dr. P. F. Whitehead, Dr. Potts, Dr. Bichfeldt, Dr. Fisher, Dr. Hoppoldt.

New Orleans.—Dr. B. A. Bobo, Dr. J. G. Byrne, Dr. Herndon.

Canton, Miss.—Dr. N. McGee, Dr. M. J. McKee, Dr. A. F. Cage.

Holly Springs.—Dr. F. M. Tennell.

Chattanooga.—Dr. E. N. Baird, Dr. R. N. Barr.

